# Echo2Pheno: a deep-learning application to uncover echocardiographic phenotypes in conscious mice

**DOI:** 10.1007/s00335-023-09996-x

**Published:** 2023-05-23

**Authors:** Christina Bukas, Isabella Galter, Patricia da Silva-Buttkus, Helmut Fuchs, Holger Maier, Valerie Gailus-Durner, Christian L. Müller, Martin Hrabě de Angelis, Marie Piraud, Nadine Spielmann

**Affiliations:** 1 Helmholtz AI, Helmholtz Zentrum München, Neuherberg, Germany; 2 Institute of Experimental Genetics, German Research Center for Environmental Health, Neuherberg, Germany; 3 Institute of Experimental Genetics, German Mouse Clinic, German Research Center for Environmental Health, Ingolstädter Landstr. 1, 85764 Neuherberg, Germany; 4 Institute of Computational Biology, Helmholtz Zentrum München, Neuherberg, Germany; 5 Department of Statistics, LMU München, Munich, Germany; 6 Center for Computational Mathematics, Flatiron Institute, New York, USA; 7 Chair of Experimental Genetics, TUM School of Life Sciences, Technische Universität München, Freising, Germany; 8 German Center for Diabetes Research (DZD), Neuherberg, Germany

## Abstract

Echocardiography, a rapid and cost-effective imaging technique, assesses cardiac function and structure. Despite its popularity in cardiovascular medicine and clinical research, image-derived phenotypic measurements are manually performed, requiring expert knowledge and training. Notwithstanding great progress in deep-learning applications in small animal echocardiography, the focus has so far only been on images of anesthetized rodents. We present here a new algorithm specifically designed for echocardiograms acquired in conscious mice called Echo2Pheno, an automatic statistical learning workflow for analyzing and interpreting high-throughput non-anesthetized transthoracic murine echocardiographic images in the presence of genetic knockouts. Echo2Pheno comprises a neural network module for echocardiographic image analysis and phenotypic measurements, including a statistical hypothesis-testing framework for assessing phenotypic differences between populations. Using 2159 images of 16 different knockout mouse strains of the German Mouse Clinic, Echo2Pheno accurately confirms known cardiovascular genotype–phenotype relationships (e.g., Dystrophin) and discovers novel genes (e.g., CCR4-NOT transcription complex subunit 6-like, *Cnot6l,* and synaptotagmin-like protein 4, *Sytl4*), which cause altered cardiovascular phenotypes, as verified by H&E-stained histological images. Echo2Pheno provides an important step toward automatic end-to-end learning for linking echocardiographic readouts to cardiovascular phenotypes of interest in conscious mice.

**Graphical abstract:**

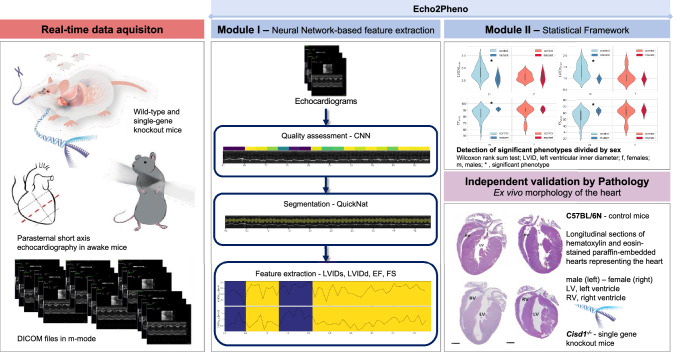

**Supplementary Information:**

The online version contains supplementary material available at 10.1007/s00335-023-09996-x.

## Main

Cardiovascular diseases are the leading cause of morbimortality in developed countries (Virani et al. 2020). Their physiology is based on the interplay of multiple genes, metabolic processes, and the environment, considerably increasing their complexity (Fuchs and Whelton 2020). Echocardiography is the most widely used imaging technique for assessing cardiac function, structure, and morphology, enabling rapid noninvasive image acquisition, screening of healthy individuals, and diagnosing complex cardiovascular diseases (Zhou et al. 2021).

Interpreting echocardiographic images is time consuming and requires specialized training. High-throughput screening rapidly collects massive amounts of data that cannot be fully evaluated manually. Only a small subset can be routinely annotated: typically, only two consecutive heartbeats in a single echocardiogram, based on the American Society of Echocardiography recommendations (Wiegers, et al. 2019). This manual expert-based analysis process is essential for meaningfully evaluating cardiac phenotypes but is critically dependent on the selection of the cardiac cycles, which is subject to high inter-rater variability, and does not exploit the complete diagnostic possibilities of the screening data. Recent advances in deep learning, which have proven successful for analyzing high-throughput data (Anaya-Isaza et al. 2021), can address these shortcomings. State-of-the-art convolutional neural networks have gained popularity for machine learning cardiovascular imaging patterns and can provide assistance with image-based diagnoses in patients (Ghorbani et al. 2020; Narang et al. 2021; He et al. 2023) and anesthetized rodent models (Grune et al. 2019; Powers et al. 2021; Duan et al. 2022). An automatic evaluation of echocardiograms recorded on conscious mice cannot be applied with the current algorithms (Grune et al. 2019; Powers et al. 2021; Duan et al. 2022) due to excessive domain shifting and is still completely missing.

Genetically modified mice are indispensable in heart research as disease models. The highly comparable heart development and structure between mice and humans are pivotal for understanding human cardiovascular disease pathogenesis (Wessels and Sedmera 2003). Over the past decade, the German Mouse Clinic (GMC, www.mouseclinic.de) has implemented an experimental setup enabling high-throughput state-of-the-art transthoracic echocardiography in mice, employing specifically designed echocardiography for acquiring mouse cardiovascular images to identify cardiac phenotypes and pathophysiological responses associated with genetic modifications (Wang et al. 2018). Here, we use this noninvasive technique to gain in vivo information about the heart of conscious animals to prevent anesthesia-related cardiac function impairment (Roth et al. 2002).

**Fig. 1:**
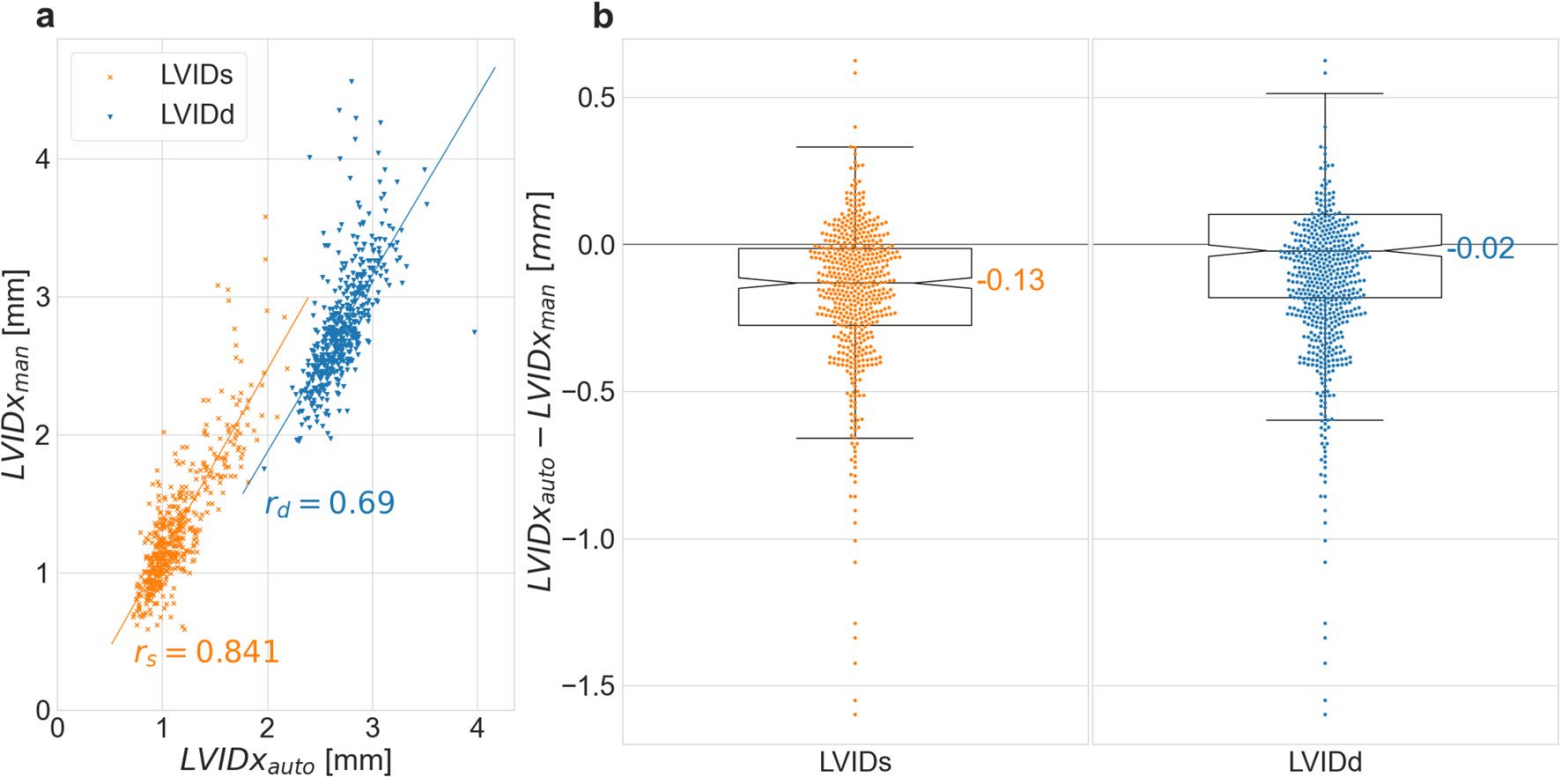
Manual versus automatic annotations of LVIDd and LVIDs in the 16 validation studies. **a** Scatter plot of LVIDx_auto_ and LVIDx_man_ and Pearson correlation coefficients (*r*_d_ $=0.69$ and *r*_s_ $=0.84$). **b** Box plots of the difference LVIDx_auto_-LVIDx_man_

To accelerate and bring new insights to our research, we developed Echo2Pheno, a novel statistical learning framework, for automatic, accurate phenotypic feature extraction from echocardiograms gathered in conscious mice “ (Figure.1 and "Data collection & datasets preparation”). Echo2Pheno consists of two modules: Modul 1, two neural networks for automatic echocardiographic image quality assessment, trace segmentation, and feature extraction and Modul 2, a statistical hypothesis-testing framework to assess phenotypic differences across subpopulations as outlined in the graphical abstract.

This work examines the Echo2Pheno framework’s ability to determine phenotypic differences in mice across genetic knockouts, based on transthoracic, parasternal, short-axis, and M-mode echocardiograms. The first module extracts parameters from the temporal traces of the left ventricle (LV) of the identified “high-quality” video recordings, i.e., from a multitude of end-systolic and -diastolic states, two key phenotypic characteristics in cardiovascular diagnostics. The goal of this study is to assess potential differences in cardiac phenotypes between wild-type and single-gene knockout mice. For that, Echo2Pheno incorporates a statistical nonparametric hypothesis-testing module with equal numbers of males and females.

Developed on 1157 echocardiography images from mutant and control mice, and validated on 2111 images from 16 independent knockout mouse strains of the GMC, Echo2Pheno enables automated verification of known gene-heart associations (e.g., dystrophin, *Dmd* gene) and facilitates the discovery of protein-coding genes yielding new insights into their potential involvement in cardiac functions. Among them, there are the CCR4-NOT transcription complex subunit 6-like (*Cnot6l*) and synaptotagmin-like protein 4 (*Sytl4*), which could be independently substantiated by heart histology where sex-specific significant changes in phenotypes were detected. Our fully automatic analysis with the Echo2Pheno framework and its benchmarking of mouse data may reveal new diagnostic possibilities, helping the discovery of novel and unexplored cardiac phenotypes and laying the groundwork for further investigation of previously unused echocardiography data in mice, even potentially extending to applications in human cardiology. It additionally opens avenues to speed up analysis in high-throughput screenings and to improve quality management thus complement previous AI-based mouse studies.

## Results

### Automatic feature/parameter extraction

#### Quality assessment and LV border detection

Automatic image quality assessment relies on a binary classifier (high/low quality), which was trained on 850 processed, manually annotated digital imaging and communications in medicine (DICOM) images in M-Mode obtained in parasternal short axis at mid-papillary level, achieving 95.4% test accuracy (F1-score 0.964; false-positive rate, 0.061; false-negative rate, 0.043) on the retained 307 test images (see Figure S1). An independent validation dataset from the 16 validation studies, including 2111 images from 517 mice, comprised 49.86% ± 7.19% high-quality images. The highest image quality was 62.25% *(*in *Acnat2* line) and lowest was 38.16% *(in Zfp280d line*).

In addition, an LV border detection network was trained on 457 images with manual LV trace annotation in only high-quality regions, achieving a dice similarity coefficient (DSC) of 0.95 and mean-squared error (MSE) of 0.02 on the test set (46 images). LV internal diameter (LVID) at end-diastole (LVIDd) and end-systole (LVIDs) extraction for each heart cycle in the “high-quality” region (i.e., the trace’s local minima and maxima) of the independent validation set led to 56,985 measurement pairs. The mean number of measurement pairs per mouse was 110 ± 78, approximately 55-fold higher than that of the manual setting (2 ± 0).

#### Manual versus automatic annotations

Figure 1a shows the correlation *r* between the average LVIDx_auto_ and LVIDx_man_ per mouse, (the automatically and manually extracted LVIDx, respectively), revealing medium (*r*_d_ = 0.69) and high mean correlations (*r*_s_ = 0.84) for LVIDd and LVIDs, respectively. The correlation per validation study is presented in Figure S2. For LVIDs, *Acnat2* had the highest correlation (*r* = 0.933, *p* < 0.001) and *Sytl4* the lowest (*r* = 0.637, *p* < 0.001). Six studies *(Acnat2, Cisd1, Dnajb14, Ergic2, Gatb, Gstm1*) showed a high Pearson correlation above the mean, whereas ten showed a medium correlation below the mean *(Cmas, Cnot6l, Dmd, Echs1, Fabp2, Kansl1l, Slc6a15, Sytl4, Uggt2, Zfp280d).* For LVIDs, all studies showing differences between manual and automatic phenotype predictions had only a medium correlation below the mean. For LVIDd, the highest correlation was in *Gstm1* (*r* = 0.929, *p* < 0.001) and lowest in *Kansl1l* (*r* = 0.424, *p* = 0.01). Ten studies showed high correlation above the mean *(Acnat2, Cmas, Cnot6l, Dnajb14, Echs1, Gatb, Gstm1, Slc6a15, Uggt2, Zfp280d*); however, six studies showed only a low-medium Pearson correlation below the mean *(Cisd1, Dmd, Ergic2, Fabp2, Kansl1l, Sytl4).*

**Fig. 2:**
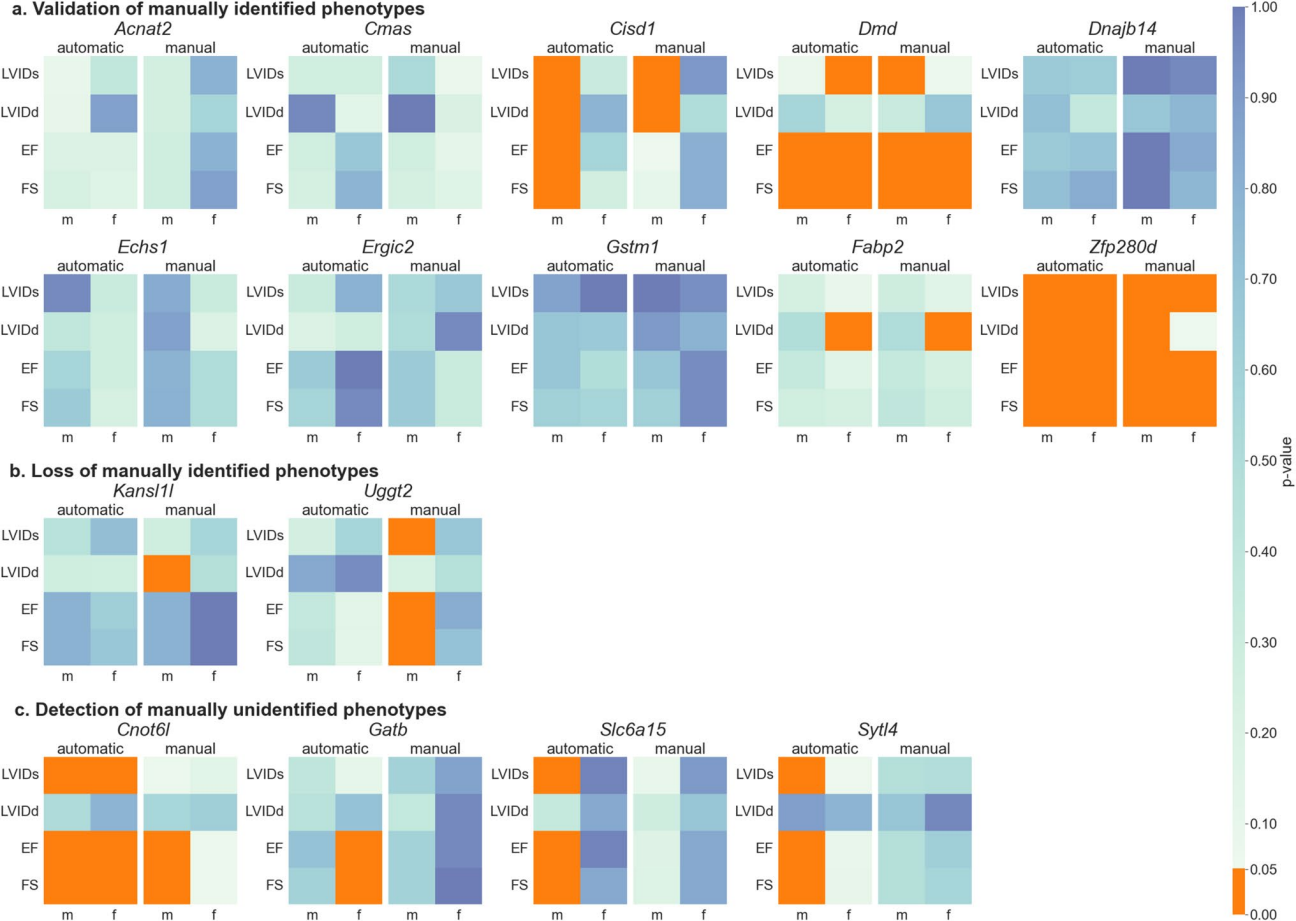
Phenotype detection with Echo2Pheno compared to the manual analysis in the 16 validation studies. Wilcoxon-Rank-Sum-Test performed  p-values for left ventricular inner diameter in systole (LVIDs), left ventricular inner diameter in diastole (LVIDd), ejection fraction (EF), and fractional shortening (FS) in male and female mice and classified the studies in **Panel** **a** Validation of manually identified phenotypes; **Panel** **b** Loss of manually identified phenotypes and **Panel** **c **Detection of manually unidentified phenotypes.

Figure 2b shows the absolute difference $(LVID{x}_{auto}-LVID{x}_{man})$ between the parameters generated by the two methods,  where the median was as small as −0.02 mm for LVIDd and −0.13 mm for LVIDs. Per study, the median differences ranged from −0.07 mm *(Cmas*) to −0.09 mm *(Kansl1l*) for LVIDd and from −0.23 mm *(Zfp280d*) to −0.05 mm *(Acnat2*) for LVIDs. This small but negative deviation indicates that Echo2Pheno generally underestimates LVIDx compared with manual annotations (Figure S3). The average mean differences of −$0.007\pm 0.30$ mm for LVIDd and −$0.174\pm 0.26$ mm for LVIDs support these hypothesis as well as the Bland–Altman analysis (see Figures S4 and S5).

### Phenotype detection with Echo2Pheno

#### Validation of manually detected phenotypes

**Fig. 3:**
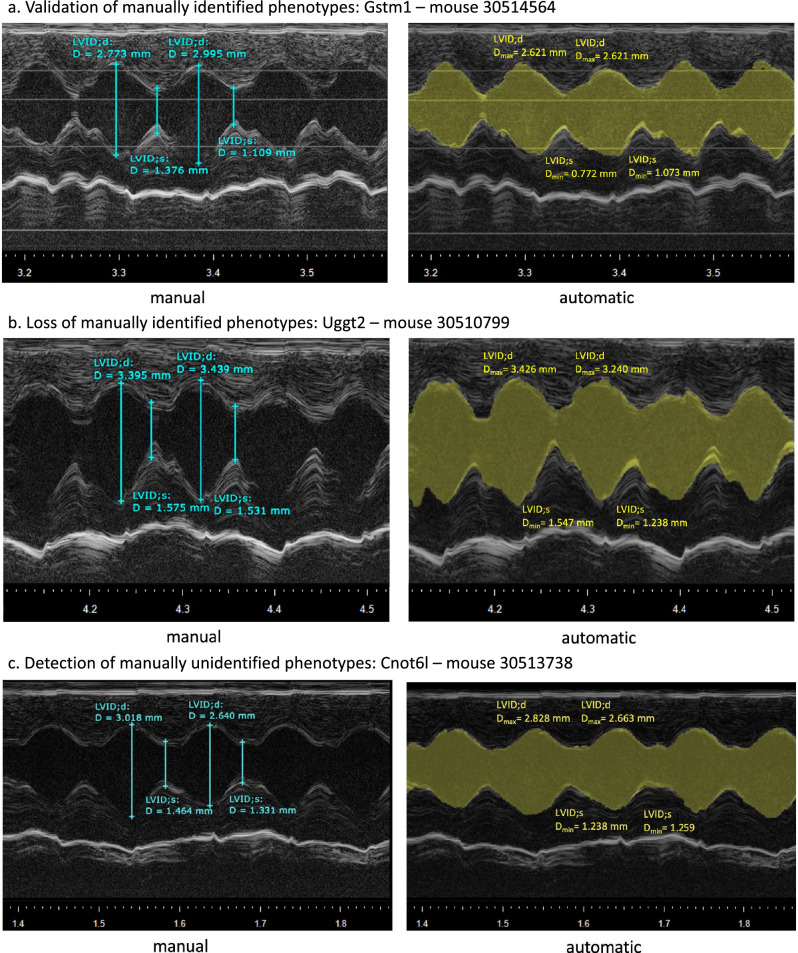
Representative echocardiograms (Visual Sonics, Vevo2100, short axis, M-mode) for comparison of manually and automatically extracted features. Manual annotation shown in the left panel is based on only two consecutive heartbeats whereas automatic Echo2Pheno-based annotation is considering the entire dataset. **Panel a** Validation of manually identified phenotypes by an agreement of manual (left panel) and automatic (right panel) analysis in *Gstm1*; **Panel b** Loss of manually identified phenotypes in LVIDs, EF, and FS due to a mismatch between manual (left) and automatic (right) analysis in *Uggt2*; **Panel c** Detection of manually unidentified phenotypes in *Cnot6l* (LVIDs for both sexes) not found with the manual annotations

Figure 2 and Table S1 show the  p-values per study, obtained based on the automatic and the manual feature comparison between the mutant and control populations. Six insignificant studies were validated as such (*Acnat2*, *Cmas*, *Dnajb14*, *Echs1*, *Ergic2*, *Gstm1*) with a representative example for *Gstm1* in Fig. 3a. In these cases, the functional parameters (LVIDs and LVIDd), as well as ejection fraction (EF), and fractional shortening (FS), derived from the functional parameters, were not significant (*p* > 0.05) in both evaluation procedures, manually and automatically, by Echo2Pheno.

A significant p-value was observed in *Cisd1* males with LVIDs_auto_ (*p* = 0.002) and LVIDd_auto_ (*p* = 0.004), validating the manual values LVIDs_man_ (*p* = 0.013), LVIDd_man_ (*p* = 0.001), as well as a gained significance in EF_auto_ (*p* = 0.007) and FS_auto_ (*p* = 0.009). In *Dmd*, significance was met in EF_auto_ females (*p* = 0.006) and males (*p* = 0.016), and in FS_auto_ females (*p* = 0. 004) and males (*p* = 0.002), thus, confirming the manual findings with EF_man_ females (*p* = 0.027) and males (*p* = 0.003), and FS_man_ females (*p* = 0.034) and males (*p* = 0.003). Moreover, the female mice gained significance in LVIDs_auto_ (*p* = 0.034), whereas LVIDs_man_ (*p* = 0.007) in males increased to LVIDs_auto_ (*p* = 0.082), falling short of the significance level. Significant manual finding validation was achieved for *Fabp2* with LVIDd_man_ (*p* = 0.018) and LVIDd_auto_ (*p* = 0.019). Significance level was reached in the automatic analysis for the whole panel of parameters in *Zfp280d* with LVIDs_auto_ (females *p* < 0.001; males *p* < 0.001), LVIDd_auto_ (females *p* = 0.019; males *p* = 0.049), EF_auto_ (females *p* = 0.002; males *p* < 0.001), and FS_auto_ (females *p* = 0.001; males *p* < 0.001). Female *Zfp280d* mutant mice, therefore, gained significance in LVIDd_auto_ (*p* = 0.019) and achieved significance in all study parameters.

#### Loss of manually found phenotypes

Significance was lost between manual and automatic in two studies: *Kansl1l* and *Uggt2* (see Fig. 2b and 3b for a representative example of *Uggt2*)*.* The extracted parameters in the male-mutated mouse population *Kansl1l (*hereinafter, mutants) were significantly different from the controls in LVIDd_man_ (*p* = 0.024); in the automatic approach, however, this significant finding was lost with LVIDd_auto_ (*p* = 0.279). Moreover, three parameters were significant in *Uggt2* manual annotation: LVIDs_man_ (*p* = 0.035), EF_man_ (*p* = 0.027), and FS_man_ (*p* = 0.021), which were lost with the automatic Echo2Pheno analysis. If the automated approach is assumed to be correct, the previous manual results can be refuted by including more echocardiographic data for deriving the parameters.

#### Detection of manually unidentified phenotypes

A significant difference (*p* < 0.05) between the mutant and control mice in the Echo2Pheno approach, which was not found with the manual annotations, suggests the detection of a manually unidentified phenotype.

Four studies gained significance in several parameters when analyzed by Echo2Pheno (Fig. 2c) with a representative example for *Cnot6l* in Fig. 3c*.* In the *Cnot6l* mutants, Echo2Pheno gained significance in LVIDs_auto_ for females (*p* = 0.031), resulting in significant EF_auto_ (*p* = 0.014) and FS_auto_ (*p* = 0.021). For the *Cnot6l* males, significance was gained in LVIDs_auto_ (*p* = 0.009), whereas EF_man_ (*p* = 0.021) and FS_man_ (*p* = 0.021) were confirmed by Echo2Pheno, both with strong *p*-values (EF_auto_ *p* < 0.001; FS_auto_ *p* < 0.001). Strictly speaking, the EF and FS of the male mutants would, therefore, belong in the “validation of manually detected phenotypes” category. Female *Gatb* became significant in EF (EF_auto_ *p* = 0.030 vs. EF_man_ *p* = 0.967) and FS (FS_auto_ *p* = 0.030 vs. FS_man_ *p* = 1.000); the males remained not significant. Male *Slc6a15* mice showed significance with Echo2Pheno in LVIDd_auto_ (*p* = 0.010), EF_auto_ (*p* = 0.007), and FS_auto_ (*p* = 0.012), whereas *Sytl4* male mice showed significance with Echo2Pheno in LVIDs_auto_ (*p* = 0.003), EF_auto_ (*p* = 0.003), and FS_auto_ (*p* = 0.007).

### Independent histopathological evaluation

**Fig. 4:**
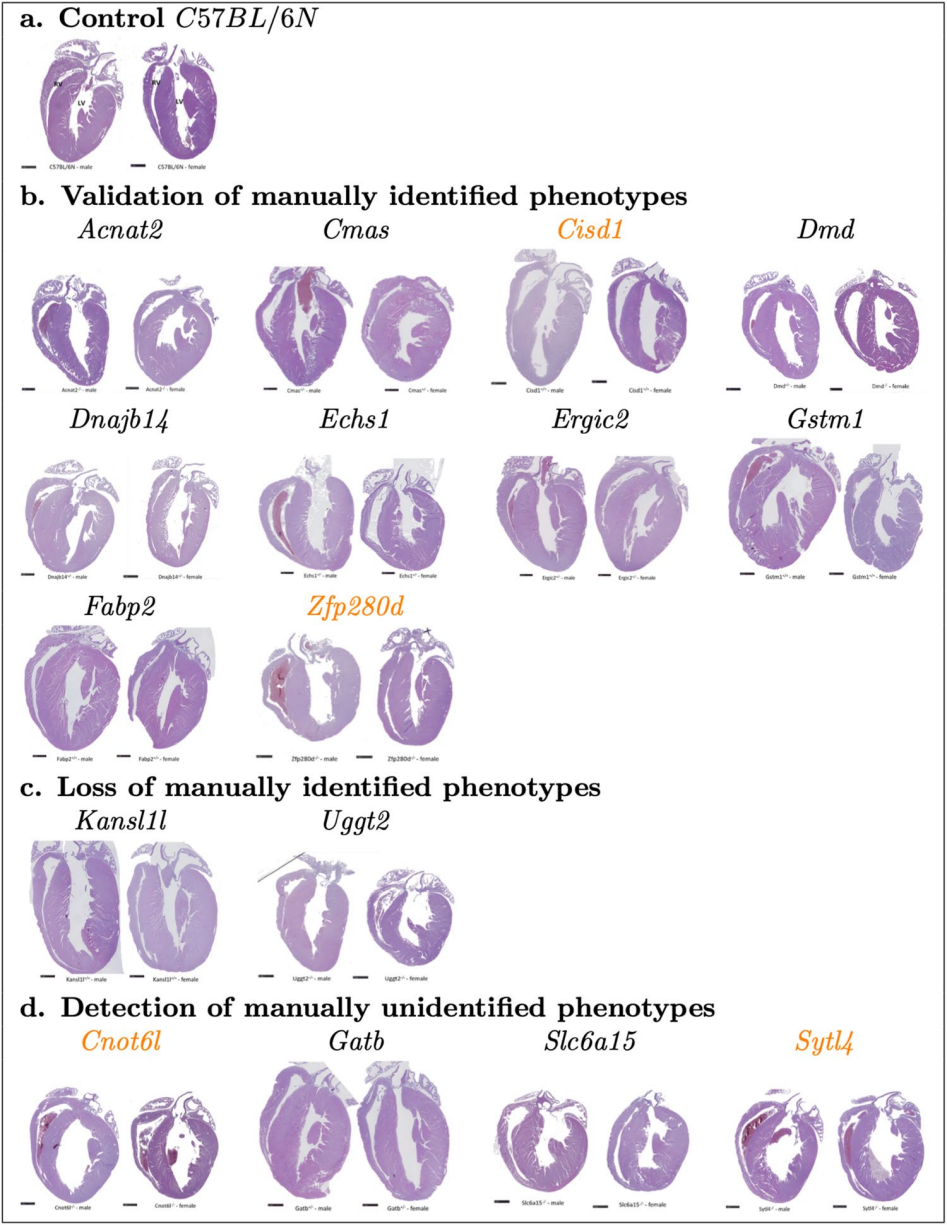
Histopathological evaluation in the 16 validation studies. Representative photomicrographs of hematoxylin and eosin-stained, paraffin-embedded longitudinal heart sections illustrating left (LV) and right (RV) ventricles. **Panel a** shows a representative example of a structurally healthy heart with normal LV dimensions in a male (left) and female (right) C57BL/6N mouse, followed by all 16 mouse validation studies **Panels b**–**d**. Studies color coded in orange show LV dilation

Figure 4 illustrates the conclusions of the independent histopathological evaluation. Six insignificant studies when manually annotated were validated by Echo2Pheno (*Acnat2, Cmas, Dnajb14, Echs1, Ergic2, Gstm1*). In those cases, histological examination revealed structurally normal hearts in all studies (Fig. 4b), confirming our findings. Four manually derived significant studies were validated as significant with Echo2Pheno (*Cisd1, Dmd, Fabp2*, *Zfp280d*)*.* One quarter of the *Cisd1* mutants showed moderate LV dilation, whereas the *Dmd* mutants had no LV alteration but focal myocardial inflammation, supporting the EF and FS alterations in this study. The *Fabp2* hearts were normal, whereas two of the four examined *Zfp280d* male mutants had dilated LVs, with inflammatory infiltrates, fibrosis, and necrotic foci in one male mutant. The histology of the heart studies that lost significance (*Kansl1l, Uggt2)* showed no abnormalities (see Fig. 4c). Ultimately, there were four studies of high interest (*Cnot6l, Gatb, Slc6a14*, *Sytl4*) with a gain of yet undiscovered significance when analyzed by Echo2Pheno. *Cnot6l* and *Sytl4* histologically exhibited enlarged LVs, with low penetrance while *Gatb* and *Slc6a15* did not. Heart section staining, however, showed signs of pericarditis by signs of fibrosis found in the pericardium when stained with sirius red in 1/4 female *Gatb* mutants.

## Discussion

Echo2Pheno saves time and resources equivalent to previously reported AI-based echocardiography tools in anesthetized rodents (Grune et al. 2019; Powers et al. 2021; Duan et al. 2022), but unsurpassed in studies with non-anesthetized rodents. It *can* annotate a mouse’s entire dataset and extract the features within a minute, whereas manual annotation, based on only two consecutive heartbeats, takes 10 times longer on average. This efficiency is in conscious mouse models, to our knowledge, unique and cannot be approximated manually.

Deep-learning models are popular in medical and preclinical echocardiography image analysis (Ghorbani et al. 2020; Narang et al. 2021; Grune et al. 2019; Powers et al. 2021; Duan et al. 2022) but all with the challenge that the algorithms are very specifically trained and not generally applicable. In mouse models, echocardiograms from anesthetized mice show excess domain shift compared to those acquired from non-anesthetized ones, making the algorithms not transferable to all echocardiography datasets. There is a meaningful proportion of scientists including the GMC who prefer conscious echocardiographic diagnostics over anesthesia to avoid known anesthetic-induced impairment of the heart (Roth et al. 2002). So far, there is no capability for this set of conscious data than the Echo2Pheno tool newly developed here, which is precisely as such, why this study is so important to fill the gap in modeling deep learning in non-anesthetized mouse models.

The reported Pearson correlation between the manual annotations and mean automatic LVIDd/LVIDs measurements per mouse indicates a good correlation, with acceptable variations. LVIDd and LVIDs showed a small but systematic shift from zero, indicating that Echo2Pheno generally extracts smaller LVIDx than manual annotations, an underestimation that could occur because 1) the human bias in selecting two heartbeats tends to focus on “broader” regions in the echocardiogram, which results in larger values in manual LV cavity measurements than the average captured by Echo2Pheno, and 2) the quality assessment has a false-positive rate of 0.118, which could contribute to this small underestimation. Minimal deviation in the standard transducer position (i.e., low-quality image) during acquisition typically causes the appearance of superimposed structures, such as the papillary muscles. In this case, the segmentation network tended to generate smaller traces since it was not trained to distinguish between superimposed structures and the true ventricular wall.

However, comparisons were performed for the mean measurements of each mouse derived from two heartbeat cycles in the manual annotations, potentially leading to a sampling bias, and a much larger number of heartbeats for Echo2Pheno*.* This much larger data pool could lead to questioning the current “ground truth,” suggesting that two heart cycles might be insufficient for obtaining instructive mouse features leading to phenotypic discovery. Nevertheless, the high correlation between manual and automatic measurements is an important indication as it validates the accuracy of our proposed framework with respect to existing pipelines.

**Fig. 5:**
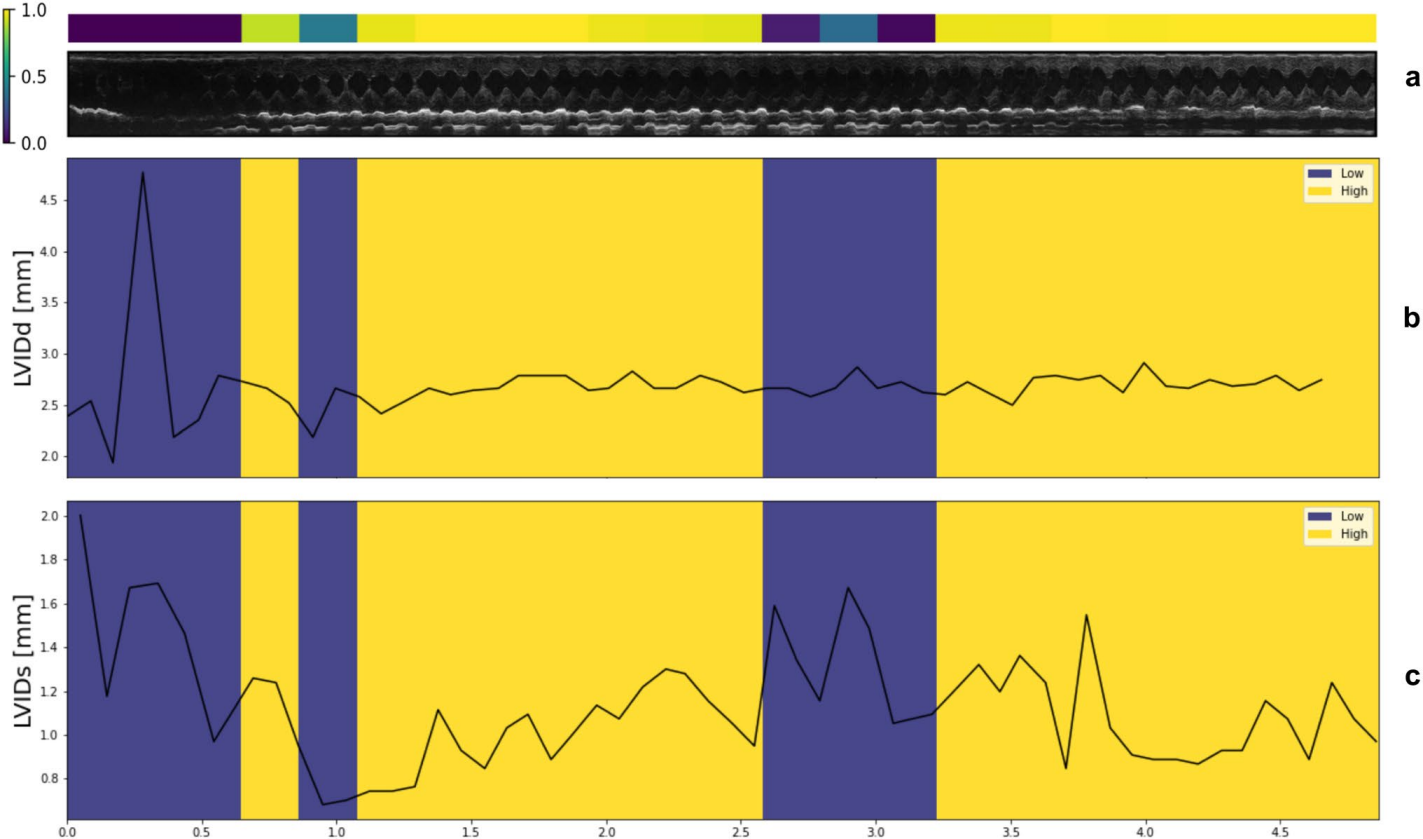
**Panel a** representative echocardiogram (Visual Sonics, Vevo2100 system) illustrating data quality labeled with color across the DICOM file. Below, yellow indicates good quality traces at a cut-off of 0.5, blue indicates low quality (**b-c**). **Panel** **b** illustrates the parameter profile of end-diastolic left ventricular inner diameter (LVIDd) and **Panel** **c** the profile of end-systolic left ventricular inner diameter (LVIDs) over recording time (ms) in one representative male *Acnat* mutant mouse

Natural physiological fluctuations with highs and lows in the data could not be captured manually until now. However, this phenomenon is present during conscious echocardiographic data acquisition (Fig. 5). LVIDs are more affected by fluctuations due to active myocardial contraction than LVIDd, which denotes cardiac relaxation. Consistent LVID values during echocardiography are therefore not guaranteed, further indicating that the current selection of two cardiac cycles is insufficient for robust diagnoses. Using all echocardiographic data can compensate for extreme values within the echocardiogram recordings due to the large amount of output measurements, consequently generating more reliable cardiac phenotypes. Indeed, of the 16 analyzed studies, Echo2Pheno matched the histopathology findings in 87.5% (14/16) cases (vs. 10/16 for manual), confirming the high accuracy of our method, which exceeds the limits of manual annotations.

For the six studies that were insignificant in both the automatic and manual evaluations *(Acnat2, Cmas, Dnajc14, Echs1, Ergic2, Gstm1*), the histopathological evaluation reported no peculiarities in heart morphology and suggest that these genes most likely do not affect the analyzed cardiac parameters. In four studies (*Cisd1, Dmd, Fabp2, Zfp280d*), the manual assessment already noted altered cardiac parameters, consolidated in diagnostics, and further substantiated by the concordant automated analysis and heart diseases for *Dmd, Zfp280d,* and *Cisd1* but not completely for *Fabp2,* wherein the hearts still appeared to be morphologically normal in the histopathological evaluation.

*CISD1,* a member of the CDGSH domain-containing family, encodes a mitochondrial outer membrane iron–sulfur protein, mitoNEET (Colca et al. 2004; Geldenhuys et al. 2019), which is an important compound in mitochondrial function/metabolism. Cardiac mitochondria isolated from mitoNEET *(Cisd1*)-null mice exhibit reduced oxidative capacity, suggesting that mitoNEET is an important iron-containing protein involved in controlling the maximal mitochondrial respiration rate (Habener et al. 2016). mitoNEET overexpression in the adipose tissue of obese/ob mice significantly reduced inflammation and oxidative stress compared with control mice (Kusminski et al. 2012). When mitoNEET was knocked out, the resulting phenotype was characterized by dopamine neurotransmitter loss in the striatum and Parkinson’s disease-type motor deficits (Geldenhuys et al. 2017). However, the detailed mechanistic aspects underlying these physiological functions remain unclear. The *Cisd1*-knockout mouse model described here showed significant LV changes, both manually and automatically, in the male mutants compared with controls. The two parameters of myocardial function (EF and FS) reached significance in male mutants only when automated by Echo2Pheno, while females showed echocardiography data comparable with the controls. For the first time, we describe an association between *Cisd1* and in vivo LV phenotypes and confirmed these results via independent cardiac histology. Male *Cisd1* hearts showed LV dilatation, although the reasons for the phenotype’s incomplete penetrance (1/2) and sexual dimorphism cannot be explained.

*DMD* encodes a large, rod-like cytoskeletal protein found at the inner surface of muscle fibers in skeletal and cardiac muscles. The most prominent mouse model to study DMD is the mdx mouse (Verhaart and Aartsma-Rus 2019; Milad et al. 2017), which shows only mild skeletal muscle histopathology, moderately affected myocardium with mild fibrosis and inflammatory cell infiltration despite the absence of dystrophin expression. Histopathological cardiac function in mdx mice, in contrast to DMD patients, is rarely observed (e.g., when very old or stressed), probably due to the large differences in muscle size, strain, regenerative capacity, and growth phases between humans and mice. Echocardiography data from our 12-week-old *Dmd-*single-gene knockout mutants (manually and automatically validated) showed no changes in LV dimensions relative to controls. However, there were altered myocardial capacities represented by EF and FS in the *Dmd* mutants. Independent histopathological examinations of the hearts confirmed our echocardiography results, given the lack of changes in LV morphology; the mutants and controls were comparable. Nevertheless, inflammatory infiltrates, fibrotic lesions, and necrotic foci were observed in the myocardial tissue of all examined  *Dmd* mice. These degenerative processes are consistent with other   *Dmd* mouse models (Verhaart and Aartsma-Rus 2019; Milad et al. 2017) and demonstrate our phenotype detection’s accuracy. Hence, the results of our *Dmd* knockout model agree with existing dystrophin mouse models and could be considered a valid proof-of-concept model for the automatic approach.

The fatty acid-binding protein 2 (*Fabp2*) was the only mouse study wherein cardiac histology revealed no changes, which is unsurprising given that the in vivo phenotype of this study was based on only one parameter (LVIDd) and sex (female) relative to control mice. A unique phenotype needs not to be a false positive; rather, such a moderate phenotype is difficult to detect in histology because the morphological changes are (still) mild during testing. *Fabp2* plays a key role in the absorption and intracellular transport of dietary long-chain fatty acids. Numerous studies have examined the association between *Fabp2* gene polymorphisms and Type 2 diabetes mellitus (Qiu et al. 2014). Several mouse models (Gajda et al. 2013) provide evidence that liver *Fabp2* plays an important role in intestinal lipid metabolism; however, there remains a lack of its association with cardiac function and morphology in the mouse model except for our data.

In contrast, the echocardiography data of *Zfp280d* mutants showed severely dilated and bilaterally confirmed (manual and automatic) LV, and thus, severe myocardial function impairment, particularly EF and FS, relative to control mice. The cardiac histology of the *Zfp280d* mutants showed enlarged LVs in the males, independently confirming our in vivo data. The zinc finger protein 280d *(Zfp280d* in mice and *ZNF280D* in humans) has not been previously associated with heart disease; we have, therefore, made a confirmatory discovery (manually and automatically). In general, *ZNF280D* is sparsely described and has no disease associations in the literature barring dyslexia (Buonincontri et al. 2011). Hence, we have undoubtedly found and described a new candidate gene with a strong link to congenital dilated cardiomyopathy.

In two studies *(Kansl1l, Uggt2*), the automatic Echo2Pheno evaluation invalidated the significant differences based on the manual evaluation. Whether they are “false positive” phenotype annotations depends on the analysis (i.e., gold standard). Interestingly, the hearts’ histopathological examination revealed no differences between the mutants and controls. Hence, the automatically generated in vivo data and morphology yielded the same results, outweighing the manual annotations. With this new type of Echo2Pheno evaluation, retrospective genotype–phenotype associations can be checked, and if necessary, corrected with reasonable effort.

The automated Echo2Pheno analysis revealed novel phenotypes in LVIDs, LVIDd, EF, and/or FS in four studies *(Cnot6l, Gatb, Slc6a15, Sytl4*). When manually annotated, one study (*Cnot6l*) had shown slight changes in EF and FS in males when manually annotated, while the remaining three were insignificant; thus, no genotype–phenotype association had previously been postulated. Echo2Pheno detected a phenodeviation in these studies. Whether they are “false negative” phenotype annotations depends on the analysis (i.e., gold standard).

*Cnot6l* had previously shown modified EF and FS in male mutants compared with controls. Presently, all (males and females) *Cnot6l* mutants had significantly altered LVIDs, EF, and FS. Histopathological examinations of the hearts confirmed our new phenotypes by altered LV morphology in *Cnot6l* mutants. To date, no human diseases have been associated with *Cnot6l* nor is its genetic influence on cardiovascular disease known. *Cnot6l* is conserved from yeast to humans (Ito et al. 2011) and maintaining its structural integrity and enzymatic activity is important for controlling cell viability (Ito et al. 2011; Horvat, et al. 2018). Our mouse data describe, for the first time, a causal association between *Cnot6l* and the heart with dilated LV.

Analysis of *Gatb* by Echo2Pheno showed new differences in EF and FS in the female mutants. Both parameters describe myocardial performance rather than the LV cavity. Accordingly, no morphological LV changes were detected in the cardiac histology; however, pericarditis was detected in 25% of the examined female hearts, which could explain the impaired pumping capacity in *Gatb* mice. Observations regarding *Gatb* mice are nonetheless interesting because of an already established link with congenital severe heart disease. Genetic defects in a subunit of the GatCAB complex, including *Gatb,* have recently been described in patients with fatal metabolic cardiomyopathy syndrome (Friederich et al. 2018). The *Gatb* knockout mouse model could contribute to the modeling and research of congenital metabolic cardiomyopathy syndrome.

*Slc6a15,* in contrast, showed significant differences in LVIDs, EF, and FS in male mutants; females remained comparable in all parameters. Histology of the heart, however, showed no LV changes in the two male hearts studied and could not support this new phenotype. The small number of hearts examined in a high-throughput screening at 16 weeks of age, the four weeks age difference between echocardiogram and pathology screens, and their arbitrary selection for histology (two out of five mutants per sex) could explain the different results. Nonetheless, this genotype–phenotype association is valuable because this gene encodes for protein family solute carrier family 6, which participates in neuronal amino acid transport, potentially associated with major depression (Kohli et al. 2011; Bröer et al. 2006). Before our report, no potential link of *Gatb* to heart disease had been described in mice.

The *Sytl4* gene is relevant to neuronal system development and is implicated in neurological and psychological diseases. *Sytl4* is most likely a candidate gene for autism (Rafi et al. 2019), but thus far, has no known association with cardiac impairment. Using Echo2Pheno*,* we found an association between *Sytl4* and cardiac impairment in male knockout mice, particularly in LVIDs, EF, and FS. Histopathologically, a dilated LV was confirmed, and myocardial fibrosis was detected. These observations provide a new causal association between *Sytl4* and the heart, possibly even myocardial remodeling.

The detection of the cardiac phenotypes of these four exemplary genes *(Cnot6l, Gatb, Slc6a15, Sytl4*) was possible only by an automated and fully comprehensive analysis of all data. The established manual method would not have attributed any significance to these genes, thereby overlooking important findings, given that three of those associations were already confirmed by histopathological findings. Although one (*Gatb*) has already been linked to heart disease in the literature, the other three phenotypes (*Cnot6l, Slc6a15, Sytl4*) describe novel findings worth further exploration.

**Fig. 6:**
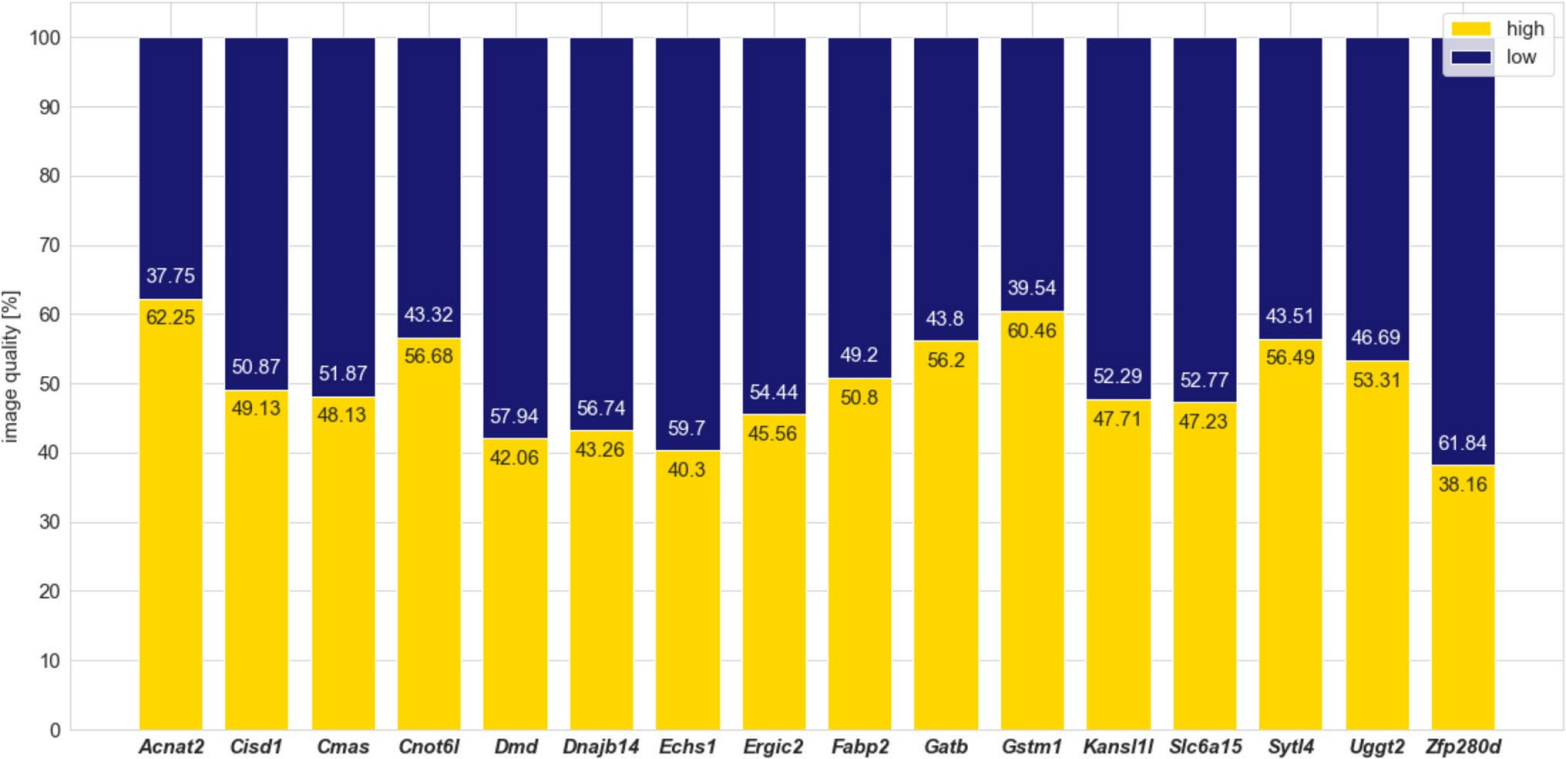
Image quality per study. The quality of the 16 different studies is presented in percentages of high-quality (yellow) and poor-quality (blue) regions

By design of high-throughput, such an automated approach does not come without limitations. Because of a particular type of echocardiography (restricted to SAX M-mode) from conscious (i.e., without sedation) 12-week-old mice with normal body weight, the Echo2Pheno algorithm is quite constrained and fails for anesthetized mice and overly dramatic phenotypes of known severe heart diseases, such as hypertrophic and dilated cardiomyopathies. Greater generalization needs to be achieved by feeding the models with larger and more diversified training data. This can occur by the incorporation of multi-centric large-scale data from the International Mouse Phenotyping Consortium (IMPC) with conscious and anesthetized echocardiography data enabling Echo2Pheno-based evaluation of all mouse echocardiographic data types. During conscious echocardiography, sudden movements and incorrect positioning of the transducer can degrade image quality, and image features may occur that do not accurately reflect the cardiac status of the mouse. On average, 49.6% of the echocardiogram data in this study did not meet our quality standards, were classified as low quality and excluded from further analysis (Fig. 6). Improving ad hoc data collection could minimize data loss. As this is a new approach designed for short-axis M-mode echocardiography of conscious mice, there is no publicly available benchmark dataset that we can use for comparison yet.

We believe that automated evaluation of all technical replicas (e.g., DICOM files) provides a new look at conscious echocardiography data and enables the extensive evaluation in all areas of the echocardiogram. The discrimination of high- from low-quality recordings provides previously impossible but important insight into the data quality and operator effect in echocardiographic data acquisition.

Echo2Pheno*,* a deep-machine-learning approach, makes high-throughput conscious M-mode SAX echocardiography data analysis fully automatic, simple, and fast. Thus, we propose Echo2Pheno-based evaluations as a state-of-the-art method for future echocardiography analysis to achieve standardization and reproducibility in conscious mouse models. This lays the foundation for overcoming the sampling bias and exploring previously unused data, while providing novel insights into gene function linked to the heart.

## Methods

### Data collection & datasets preparation

#### Echocardiography procedure in mice

Animal housing was performed in strict accordance with directive 2010/63/EU. All mice within the two barriers (experimental barrier GMC and separate breeding barrier) were housed in individually ventilated caging systems (Seal-safe plus, GM 500, Tecniplast, Buggugiate, Italy) under specific pathogen-free condition (GMC), with a maximum cage density of five adult mice per cage. Mice homozygous (or heterozygous if homozygous were lethal; Table S2 and S3) for single-gene knockouts were bred on the C57BL/6N background strain in the animal facility of the Helmholtz Zentrum München (https://www.mouseclinic.de/) (Fuchs et al. 2018). Experiments were performed according to the German laws for animal protection and by permission of the Government of Upper Bavaria.

Echocardiograms were performed on 12-weeks-old mice after allowing them to adjust to the experimental area at least 30 min before measurements, and all examinations were performed in quiet room conditions to reduce external stimuli that could interfere with mouse physiology. To eliminate circadian influences, ultrasound was performed between 8 and 11 am. Body weights were measured shortly before echocardiography.

Cardiac function was evaluated with transthoracic echocardiography using a Vevo2100 or Vevo3100 Imaging System (VisualSonics Inc., Toronto, Canada) with a 40-MHz probe and a focus depth of 0.6. For echocardiographic examinations, the mice were firmly held by the nape of the neck (in the supine position) in the palm of one hand with the tail held tightly between the last two fingers. Pre-warmed ultrasound gel was applied on the chest at the imaging location.

For accurate linear LVID measurements and LV wall thicknesses, an M-mode image of the heart in parasternal short-axis view was acquired. The transducer was rotated approximately 90° counterclockwise, starting from the parasternal long-axis view. The M-mode cursor was placed perpendicular to the interventricular septum and posterior wall of the LV at the level of the papillary muscles. At the end of an imaging session, ultrasound gel was removed with water-dampened gauze. Examinations were performed on conscious animals to prevent anesthesia-related impairment of cardiac function (Roth et al. 2002). All echocardiograms were recorded and analyzed by the same person, blinded to the mouse genotype.

The acquired images depict the LVID of the heart during contraction over time. Each record is stored as a DICOM file under the Ultrasound Multi-frame Image Storage standard and comprises 49 frames. All acquisitions have a total length of 4.869 s with a pixel resolution of 0.833 ms on the x-axis, whereas the resolution on the y-axis varies for each acquisition.

#### Datasets

##### General

Details of the datasets used in this work are given in Table S2 and S3, and summarized in Table 1. All mouse studies included at least seven male and female mutants each to ensure a minimum of 14 mice per study, amounting to a mean mutant percentage of 46.94% in the training dataset, 37.5% in the test dataset, and 52.88% in the independent validation data. All mice had normal body weights (mean 25.05 g, 22.94 g, and 24.39 g for the training, test, and independent validation groups, respectively). In both datasets, presented below, the echocardiograms satisfied the same technical prerequisites as explained in “Echocardiography procedure in mice.”

**Table 1:** Summary of dataset characteristics. No., Number; SD, standard deviation; LV, left ventricle

Characteristics	Module I		Independent Validation
	Training	Test	
No. of studies	9	5	16
No. of mice	49	8	517
No. of images (quality assessment)	850	307	2111
No. of images (LV trace segmentation)	457	46	2111
Sex (% male)	51.02	37.50	49.03
Age (weeks of life)	12	12	12
Weight: mean, g (SD)	25.05 (3.73)	22.94 (3.50)	24.39 (3.13)
Genotype (% mutants)	46.94	37.5	52.88

##### Echo2Pheno dataset

Both neural networks for Module I were trained and tested on GMC murine echocardiography data. Table 1 presents training data that originated from nine experimental studies and test images from five studies with a mixture of males and females to ensure a large amount of variability in the data. For the tasks of classifying acquisition quality and segmenting the LVID, ten and six mice were set aside, respectively, to create test sets for evaluating the performance of the networks.

In the 57 echocardiography images that were collected regions of high and low acquisition quality were defined and provided by experts. These regions had a minimum length of 0.3 s, and each acquisition could have either one or multiple high and low regions. In addition, experts annotated the LV upper and lower traces in all good acquisition regions, which served as ground truth for the segmentation task. Finally, the images were processed and split into smaller sections (see “Datasets”), resulting in a dataset of 850 images for training and 307 images for testing. This dataset was used for training and testing the quality classification network of Module I, where the training set consists of 374 high- and 476 low-quality images, while the test set consists of 209 high- and 98 low-quality images. For the segmentation network only, the high-quality regions of the echocardiograms were used, since regions of low quality do not truly represent the heart state and we aspired that the model learns true representations of the LVID, given that it is ultimately from such regions that we wish to extract features. This resulted in a dataset of 457 images for training and 46 images for testing.

##### Independent validation dataset

The independent validation dataset comprises 16 randomly chosen studies (520 mice, 2111 images), 14 of them completely unseen (Table S3). We excluded all images used in the Echo2Pheno dataset from the other two studies (*Dmd* and *Slc6a15*). Here, mutants were always matched with wild-type (control) animals of the same genetic background (C57BL/6N), and each study included equal number of males/females. Mutants were hereinafter characterized by the induced loss of a protein-coding gene name. 

##### Histopathology of the heart

Cardiac histology cannot validate functional parameters such as EF and FS, as the heart is no longer beating/pumping. Seeking for supportive information regarding cardiac dimensions, histopathology is the diagnostic of choice in the GMC. It allows identification of altered LV dimensions and myocardium changes, which may correspond to in vivo echocardiography (LVIDs/LVIDd phenotypes). Histopathology at 16 weeks of age is complementary to functional echocardiography examination (12 weeks of age) for the detection of cardiovascular phenotypes that are not always evident particularly in young mice.

Thus, high-throughput histopathological cardiac analysis was applied to all analyzed studies. Four mutant mice (two males and females) and two concurrent controls (one male and female) from each study were euthanized with CO_2_, after blood sample removal, at 16 weeks of age. The mice were analyzed macroscopically and heart weights were measured. The hearts were formalin-fixed, paraffin-embedded, sectioned through the longitudinal axis and hematoxylin and eosin stained for histological examination. The heart chambers, intraventricular septum, valves, papillary muscle, and a portion of the major vessels at the base of the heart were considered landmarks during sectioning to ensure consistent heart sampling. Stained sections were scanned with a Hamamatsu digital slide scanner (NanoZoomer, Japan). Of note, there was no possibility to control for stopping the hearts at a consistent stage of the cardiac cycle.

Figure 5 shows representative photomicrographs of longitudinal sections of hematoxylin–eosin-stained, paraffin-embedded hearts representing the left/right ventricles. For the purpose of this study, the microphotos of the stained heart sections focused on the dimensions of the LV and on abnormalities in myocardium tissue structure (lymphocytic infiltration and fibrosis). Histopathological assessment of cardiac chamber size, valves, epicardium, myocardium, and endocardium lesions in mutant and control mice was independently performed by two pathologists.

### Echo2Pheno—models & training

#### Pre-processing

To make the data accessible to neural networks, several preprocessing steps were required. Given that an original echocardiography image consists of overlapping periods, we first processed the frames to acquire one continuous image corresponding to the full time of acquisition with unique regions. The start and end times of high and low image quality (provided annotations) were then used to split the image into smaller parts, resulting in smaller images with varying length and size. We, therefore, further cropped our data by using a sliding window approach with a fixed step. In this way, square images of size 256 × 256 were extracted, depicting the interior of the LV and comprising approximately three heartbeats, since this is typically the area investigated by human experts during manual annotation. For the LVID segmentation task, the network needs to see the full height of the image, in order to learn to distinguish the LVID. We, therefore, extracted images including the full height of the original images while again applying a sliding window approach. With the annotated upper and lower traces in the high-quality images, we created binary segmentation masks that were used as ground truth during training and testing.

#### Echo2Pheno Module I

Echo2Pheno’s Module I is designed to automatically estimate functional parameters in transthoracic short-axis echocardiograms of conscious mice. During experiments, sudden movements and transducer misplacement can deteriorate acquisition quality and image features that do not truly represent the mouse’s cardiac state can appear. Two independent steps were, therefore, performed: (1) image quality classification using a convolutional neural network and (2) LV trace segmentation by an adapted QuickNat architecture (Roy et al. 2019), with subsequent extraction of the functional parameters (LVIDs, LVIDd, EF and FS), averaged over the high-quality echocardiogram regions for each mouse.

##### Image quality assessment

The quality classification network architecture was designed to be simple and efficient, including five convolutional blocks (Convolution—ReLU—BatchNorm—MaxPooling) followed by a fully connected layer and a sigmoid function. The convolutional filters and max pooling layers have a kernel size of three and two, respectively. The network was trained for 15 epochs, using cross entropy loss and the Adam optimizer with a learning rate of 1e-04 (Kingma and Ba 2014).

##### Segmentation

For the LVID segmentation task, the QuickNAT model architecture was adjusted for a binary output. QuickNAT follows and extends the U-Net architecture (Ronneberger et al. 2015), with additional internal skip connections included in each dense block. It consists of four such dense blocks in the encoder and decoder, accepts inputs of any size and was chosen due to its inference speed and proven high performance in medical imaging segmentation tasks. The segmentation network was trained with the same conditions as the classification network for 20 epochs.

##### Feature extraction and filtering

From the binary masks produced by the segmentation network, we measured the LVID for each time instance, corresponding to the rows and columns of the image, respectively. Next, we searched for local minima and maxima, thereby obtaining all occurrences of the systole and diastole phases, namely LVIDd and LVIDs. The time of their incidence was also stored. During validation, we used the quality assessment output to filter for all good pairs of LVIDd and LVIDs. Furthermore, we ensured that LVIDd is larger than LVIDs within one pair. With the filtered pairs, we then used the Teichholz formula (Arora et al. 2010) to compute EF and FS:

$$\begin{gathered} EF = \frac{LV\,VOLd - LV\,VOLs}{{LV\,VOLd}} \times 100\,and\,FS = \frac{LVIDd - LVIDs}{{LVIDs}} \times 100, \hfill \\ \,where\,LV\,Volx = \frac{7}{2.4 + LVIDx} \times LVIDx^{3} ,x \in \left\{ {s,d} \right\} \hfill \\ \end{gathered}.$$

Thus, for each good heartbeat corresponding to one LVIDd–LVIDs pair, four features were extracted along with their time of incidence: LVIDd and LVIDs in mm, EF in %, and FS in %. Finally, for each mouse, the average across all heartbeats in high-quality regions of these features was obtained.

#### Echo2Pheno Module II

Given that our aim was to explore a genotype–phenotype difference, Module II of Echo2Pheno investigated whether the mutant and control measurements of a study were drawn from the same statistical distribution. The parameters that characterized the LV cavity were compared between mutants and controls to indicate gene function in terms of cardiac (LV) contractility/dimension. We, therefore, performed a two-sided Wilcoxon rank-sum test for all four parameters extracted from Module I to compare these two populations. P-values were extracted and stratified by sex for LVIDd, LVIDs, EF, and FS. A threshold of 0.05 was set on the outcome of the tests to define whether the control and mutant populations were significantly different for each parameter.

#### Data availability

The Echo2Pheno framework, along with the code for training and testing the classification and segmentation models, is freely available at https://github.com/HelmholtzAI-Consultants-Munich/Echo2Pheno for testing and applying to independent datasets.

### Evaluation: Echo2Pheno & histopathology

#### Echo2Pheno

##### Module I

The quality assessment network was evaluated using the accuracy, F1-score, and false-positive and -negative rates, while the segmentation network was evaluated using the DSC and MSE, with the final network giving a DSC of 0.95 and MSE of 0.02 on the test set. For both models, the corresponding test sets of the Echo2Pheno dataset were used.

##### End-to-end evaluation

To evaluate our framework, we calculated the p-values similarly extracted for all manual annotations and automatically extracted parameters, as reported in Table S1 and Fig. 3. Here, the independent validation dataset was used. A phenotype was considered validated when the significance of the p-values from the manually generated measurements coincided with those derived from Echo2Pheno, whereas a phenotype was lost when a significant manually computed p-value became insignificant when applying Echo2Pheno. For a correct comparison between the outcomes of the tests, we used exactly the same sample size, except in two cases: given that we filtered for only good measurements, we lost one male control sample from *Gatb* and two male mutants from *Zfp280d*.

#### Independent histopathological evaluation

In this step, we assumed LV phenotype as independently validated when altered LV dimensions were detected by cardiac histology, confirming the LVIDs/LVIDd significance after applying Echo2Pheno, whereas an LV phenotype was completely lost when the histopathological analysis was comparable between mutants and controls. Comparing the heart weights of mutant and control mice in a linear model with body weight as a covariate in the analysis revealed no significant differences except for *Dmd*, wherein the heart weight was increased in the female mutants but decreased in the male mutant mice when compared with respective controls.

## Supplementary Information

Below is the link to the electronic supplementary material.

Supplementary file1 (PDF 3818 KB)

Supplementary captions (doc 13 KB)

## Data Availability

Data availability upon request. Please contact the corresponding author.
