# Supplementary material for: Echo2Pheno: a deep-learning application to uncover echocardiographic phenotypes in conscious mice
Source: Mamm Genome. 2023 May 23;34(2):200–15. doi: 10.1007/s00335-023-09996-x (PMC10290584; doi:10.1007/s00335-023-09996-x)
Supplement: Supplementary file 1 — Supplementary file1 (PDF 3818 KB) [file 335_2023_9996_MOESM1_ESM.pdf]

Figure S1

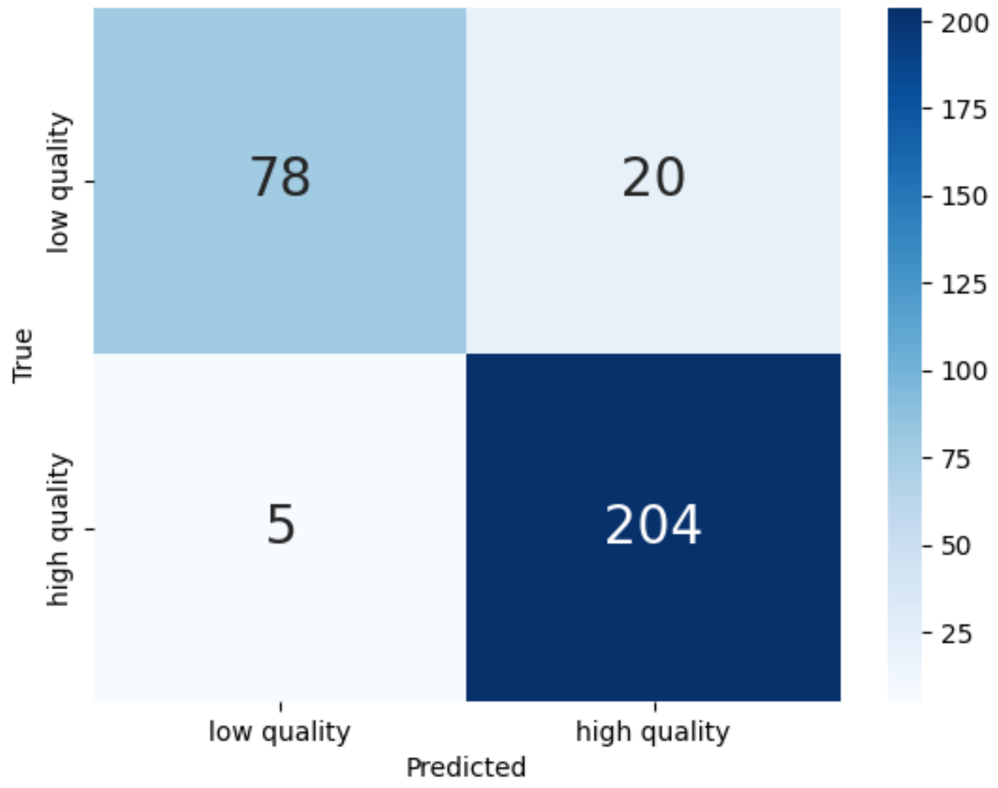

78
20
5
204
low quality
high quality
True
low quality
high quality
Predicted
200
175
150
125
100
75
50
25

Figure S2

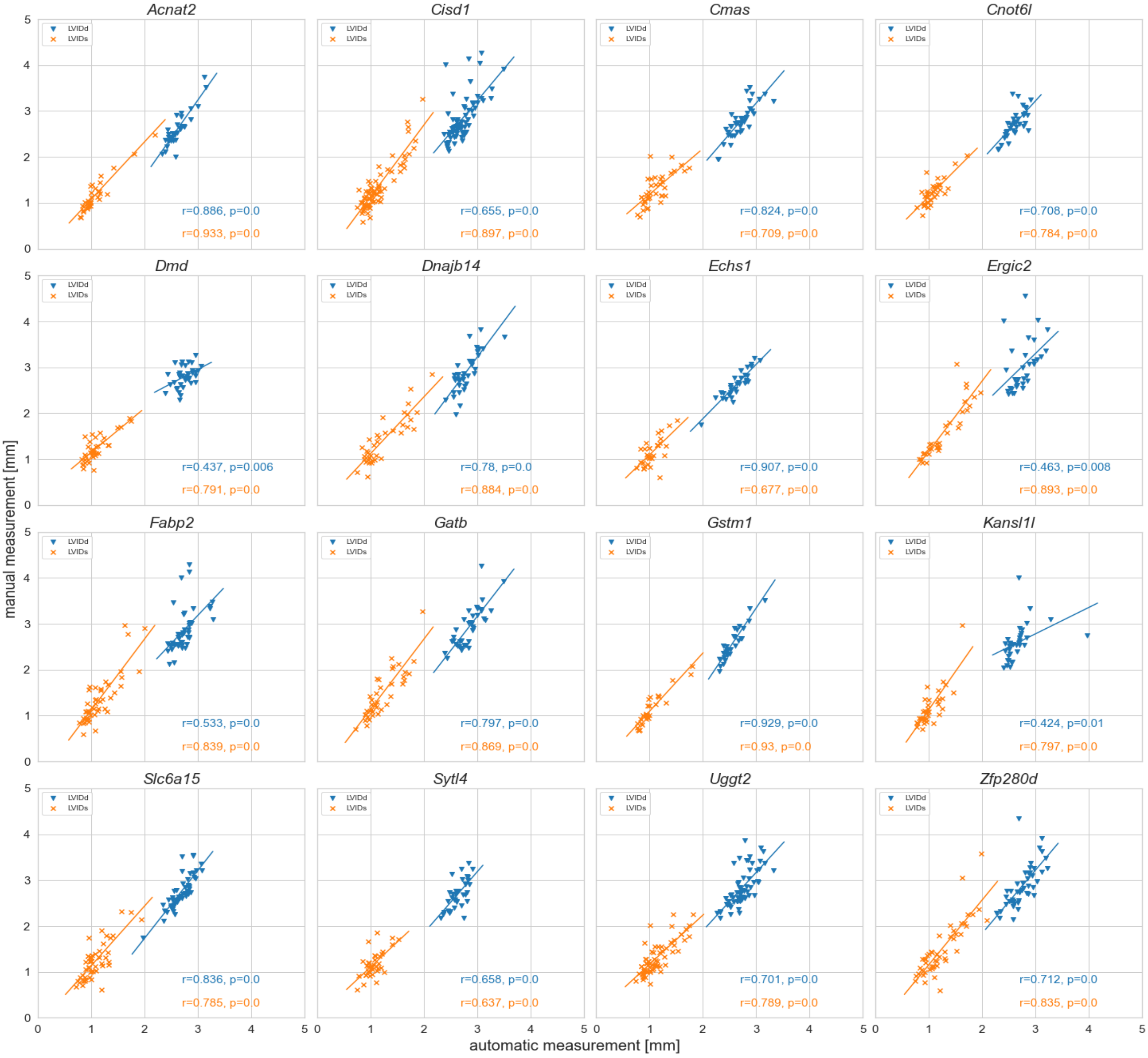
Acnat2
r=0.886, p=0.0
r=0.933, p=0.0
Cisd1
r=0.655, p=0.0
r=0.897, p=0.0
Cmas
r=0.824, p=0.0
r=0.709, p=0.0
Cnot6l
r=0.708, p=0.0
r=0.784, p=0.0
Dmd
r=0.437, p=0.006
r=0.791, p=0.0
Dnajb14
r=0.78, p=0.0
r=0.884, p=0.0
Echs1
r=0.907, p=0.0
r=0.677, p=0.0
Ergic2
r=0.463, p=0.008
r=0.893, p=0.0
Fabp2
r=0.533, p=0.0
r=0.839, p=0.0
Gatb
r=0.797, p=0.0
r=0.869, p=0.0
Gstm1
r=0.929, p=0.0
r=0.93, p=0.0
Kansl1l
r=0.424, p=0.01
r=0.797, p=0.0
Slc6a15
r=0.836, p=0.0
r=0.785, p=0.0
Sytl4
r=0.658, p=0.0
r=0.637, p=0.0
Uggt2
r=0.701, p=0.0
r=0.789, p=0.0
Zfp280d
r=0.712, p=0.0
r=0.835, p=0.0
LVIDd
LVIDs
manual measurement [mm]
automatic measurement [mm]

Figure S3

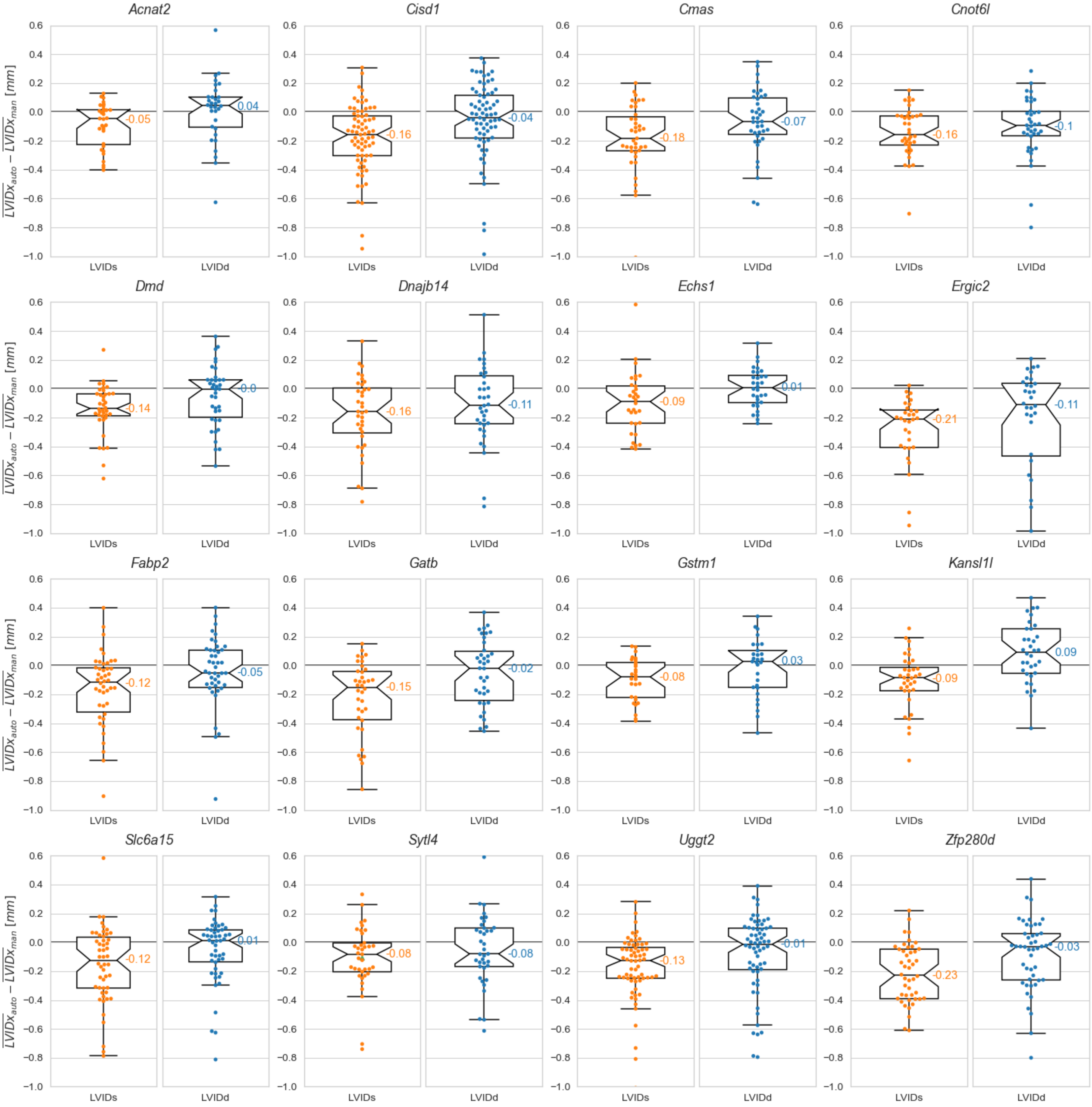
Acnat2
Cisd1
Cmas
Cnot6l
Dmd
Dnajb14
Echs1
Ergic2
Fabp2
Gatb
Gstm1
Kansl1l
Slc6a15
Sytl4
Uggt2
Zfp280d
LVIDs
LVIDd
$\overline{LVIDx_{auto}} - \overline{LVIDx_{man}}$ [mm]
0.6
0.4
0.2
0.0
−0.2
−0.4
−0.6
−0.8
−1.0
-0.05
0.04
-0.16
-0.04
-0.18
-0.07
-0.16
-0.1
-0.14
0.0
-0.16
-0.11
-0.09
0.01
-0.21
-0.11
-0.12
-0.05
-0.15
-0.02
-0.08
0.03
-0.09
0.09
-0.12
0.01
-0.08
-0.08
-0.13
0.01
-0.23
-0.03

Figure S4

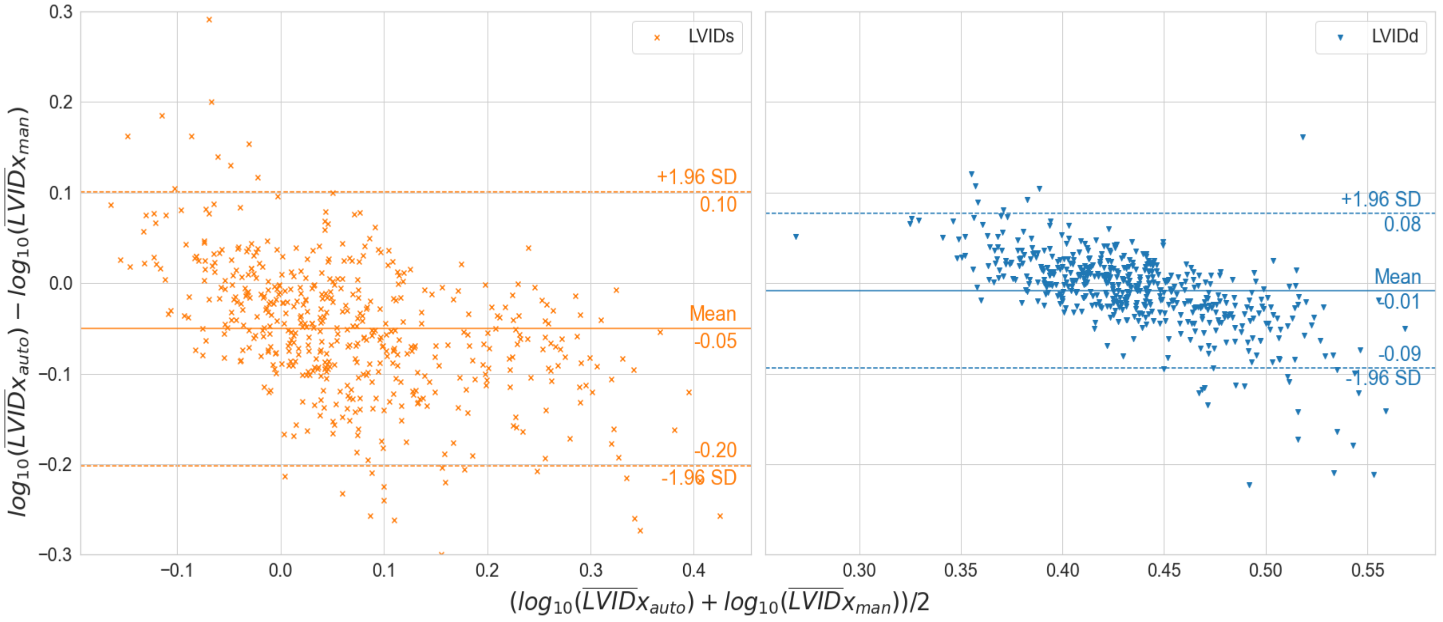

Figure S5

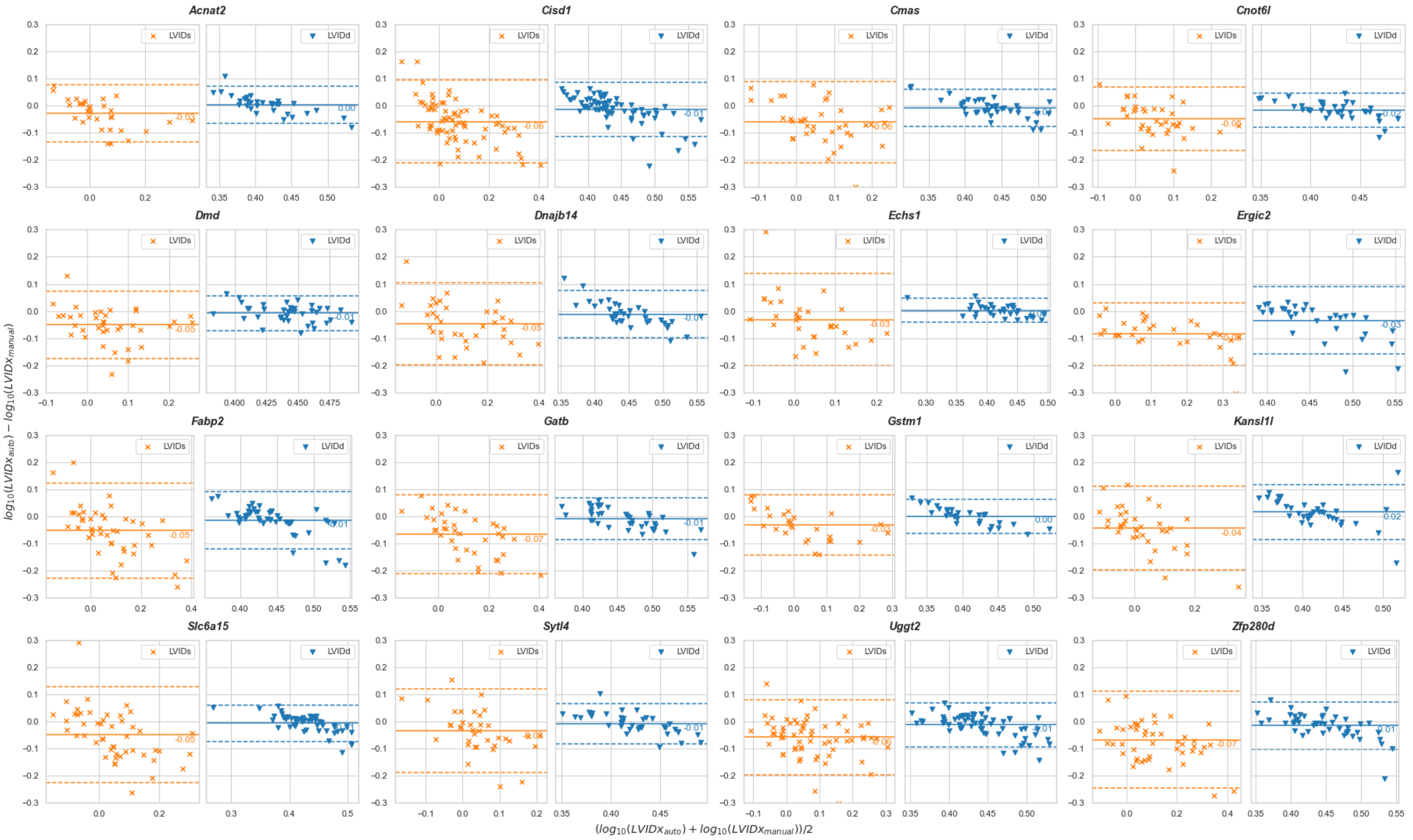

Table S1

		p			
		Automatic		Manual	
		Male	Female	Male	Female
Acnat2	LVIDs	0.083	0.382	0.248	0.798
	LVIDd	0.105	0.878	0.248	0.574
	EF	0.195	0.195	0.279	0.798
	FS	0.234	0.161	0.279	0.878
Cisd1	LVIDs	0.002	0.335	0.013	0.912
	LVIDd	0.004	0.786	0.001	0.506
	EF	0.007	0.574	0.053	0.814
	FS	0.009	0.266	0.107	0.814
Cmas	LVIDs	0.270	0.273	0.539	0.076
	LVIDd	0.967	0.140	1.000	0.212
	EF	0.270	0.678	0.270	0.121
	FS	0.236	0.791	0.236	0.162
Cnot6l	LVIDs	0.009	0.031	0.068	0.140
	LVIDd	0.515	0.791	0.563	0.623
	EF	< 0.001	0.014	0.021	0.064
	FS	< 0.001	0.021	0.027	0.064
Dmd	LVIDs	0.082	0.034	0.007	0.068
	LVIDd	0.571	0.237	0.335	0.689
	EF	0.016	0.006	0.003	0.027
	FS	0.002	0.004	0.003	0.034
Dnajb14	LVIDs	0.659	0.633	1.000	0.967
	LVIDd	0.724	0.360	0.691	0.775
	EF	0.659	0.696	1.000	0.838
	FS	0.724	0.829	1.000	0.775
Echs1	LVIDs	0.959	0.328	0.833	0.317
	LVIDd	0.382	0.279	0.878	0.195
	EF	0.574	0.279	0.793	0.505
	FS	0.645	0.234	0.798	0.505
Ergic2	LVIDs	0.328	0.798	0.528	0.674
	LVIDd	0.195	0.279	0.505	0.958
	EF	0.645	1.000	0.645	0.328
	FS	0.574	0.958	0.645	0.328
Fabp2	LVIDs	0.241	0.087	0.284	0.154
	LVIDd	0.485	0.019	0.500	0.018
	EF	0.351	0.147	0.351	0.236
	FS	0.275	0.236	0.393	0.280

		p			
		Automatic		Manual	
		Male	Female	Male	Female
Gatb	LVIDs	0.391	0.111	0.596	0.870
	LVIDd	0.438	0.713	0.369	0.967
	EF	0.713	0.030	0.596	0.967
	FS	0.596	0.030	0.596	1.000
Gstm1	LVIDs	0.867	1.000	1.000	0.948
	LVIDd	0.694	0.662	0.908	0.796
	EF	0.694	0.491	0.694	0.950
	FS	0.613	0.573	0.613	0.950
Kansl1l	LVIDs	0.442	0.734	0.279	0.570
	LVIDd	0.279	0.273	0.024	0.473
	EF	0.798	0.623	0.798	1.000
	FS	0.798	0.678	0.798	1.000
Slc6a15	LVIDs	0.010	0.977	0.095	0.910
	LVIDd	0.350	0.843	0.282	0.692
	EF	0.007	0.977	0.165	0.843
	FS	0.012	0.843	0.183	0.843
Sytl4	LVIDs	0.003	0.054	0.475	0.496
	LVIDd	0.887	0.791	0.695	0.970
	EF	0.003	0.089	0.475	0.623
	FS	0.007	0.104	0.475	0.571
Uggt2	LVIDs	0.245	0.570	0.035	0.681
	LVIDd	0.849	0.953	0.220	0.469
	EF	0.364	0.142	0.027	0.829
	FS	0.386	0.132	0.021	0.710
Zfp280d	LVIDs	<0.001	< 0.001	< 0.001	0.004
	LVIDd	0.049	0.019	< 0.001	0.061
	EF	<0.001	0.002	< 0.001	0.001
	FS	<0.001	0.001	< 0.001	0.001

Table S2

	Study	Zygosity	Background	Sex	Genotype	$bw[g]$	Use
1	***Arvcf***	homozygous	C57BL/6N	f	control	22.20	train
2		homozygous	C57BL/6N	f	control	28.10	train
3		homozygous	C57BL/6N	f	control	25.40	test
4		heterozygous	C57BL/6N	f	mutant	23.10	train
5		heterozygous	C57BL/6N	f	mutant	24.10	train
6	***Anpep***	homozygous	C57BL/6N	m	control	28.60	train
7		homozygous	C57BL/6N	m	mutant	31.70	train
8		homozygous	C57BL/6N	m	mutant	36.40	train
9	***Cenpv***	homozygous	C57BL/6N	m	control	31.70	train
10		homozygous	C57BL/6N	m	control	31.50	train
11		homozygous	C57BL/6N	f	mutant	22.20	train
12		homozygous	C57BL/6N	f	mutant	24.40	train
13	***Crygn***	homozygous	C57BL/6N	m	control	26.70	train
14		homozygous	C57BL/6N	f	control	21.30	test
15		homozygous	C57BL/6N	m	control	27.30	test
16		homozygous	C57BL/6N	f	mutant	21.20	train
17	***Dmd***	homozygous	C57BL/6N	m	control	26.90	train
18		homozygous	C57BL/6N	f	control	22.60	train
19		hemizygous	C57BL/6N	m	mutant	23.90	train
20		homozygous	C57BL/6N	f	mutant	25.00	train
21		hemizygous	C57BL/6N	m	mutant	25.70	train
22	***Slc6a15***	homozygous	C57BL/6N	f	control	26.80	train
23		homozygous	C57BL/6N	m	control	32.10	train
24		homozygous	C57BL/6N	f	mutant	25.00	train
25	***Slc25a12***	homozygous	C57BL/6N	m	control	26.80	train
26		homozygous	C57BL/6N	f	control	23.10	train
27		homozygous	C57BL/6N	f	control	21.30	train
28		homozygous	C57BL/6N	m	control	24.20	train
29		homozygous	C57BL/6N	f	control	19.40	train
30		heterozygous	C57BL/6N	m	mutant	22.20	train
31		heterozygous	C57BL/6N	f	mutant	20.30	train
32		heterozygous	C57BL/6N	f	mutant	23.60	train
33		heterozygous	C57BL/6N	f	mutant	21.80	train
34		heterozygous	C57BL/6N	m	mutant	23.30	train
35		heterozygous	C57BL/6N	m	mutant	21.70	train
36		heterozygous	C57BL/6N	m	mutant	26.70	train
37		heterozygous	C57BL/6N	f	mutant	23.10	train
38		heterozygous	C57BL/6N	m	mutant	24.40	train
39		heterozygous	C57BL/6N	f	mutant	19.50	train
40		heterozygous	C57BL/6N	m	mutant	24.20	train
41		heterozygous	C57BL/6N	f	mutant	19.80	test

	Study	Zygosity	Background	Sex	Genotype	$bw[g]$	Use
42	***Shisal2a***	homozygous	C57BL/6N	m	control	24.90	train
43		homozygous	C57BL/6N	m	control	24.90	train
44		homozygous	C57BL/6N	f	control	17.30	test
45		homozygous	C57BL/6N	f	mutant	17.90	train
46		homozygous	C57BL/6N	m	mutant	26.90	test
47	***Epigenetic***	homozygous	C57BL/6N	m	control	28.30	train
48		homozygous	C57BL/6N	m	control	28.30	train
49		homozygous	C57BL/6N	m	control	28.10	train
50		homozygous	C57BL/6N	m	control	26.60	train
51		homozygous	C57BL/6N	f	control	24.00	train
52		homozygous	C57BL/6N	f	control	21.80	train
53		homozygous	C57BL/6N	m	control	27.90	train
54		homozygous	C57BL/6N	f	control	22.30	train
55		homozygous	C57BL/6N	f	control	22.50	train
56		homozygous	C57BL/6N	f	control	23.30	test
57		homozygous	C57BL/6N	m	control	22.20	test

Table S3

	Zygosity	Background	Sex	Genotype	$\overline{bw}[g]$	No.
Acnat2	heterozygous	C57BL/6N	f	control	21.76	8
				mutant	21.65	8
			m	control	26.83	8
				mutant	27.63	8
Cisd1	homozygous	C57BL/6N	f	control	22.45	29
				mutant	22.10	8
			m	control	27.13	28
				mutant	24.64	8
Cmas	heterozygous	C57BL/6N	f	control	21.62	10
				mutant	22.26	10
			m	control	26.02	10
				mutant	27.93	9
Cnot6l	homozygous	C57BL/6N	f	control	21.84	10
				mutant	21.03	10
			m	control	27.03	10
				mutant	24.69	8
Dmd	hemi-/homozygous	C57BL/6N	f	control	21.83	10
				mutant	25.21	8
			m	control	27.16	12
				mutant	27.98	8
Dnajb14	heterozygous	C57BL/6N	f	control	22.60	9
				mutant	21.48	10
			m	control	27.33	9
				mutant	27.10	9
Echs1	heterozygous	C57BL/6N	f	control	22.31	8
				mutant	23.09	8
			m	control	27.14	8
				mutant	28.75	8
Ergic	heterozygous	C57BL/6N	f	control	22.01	8
				mutant	23.04	8
			m	control	26.66	8
				mutant	28.20	8
Fabp2	homozygous	C57BL/6N	f	control	21.93	16
				mutant	21.39	10
			m	control	26.42	13
				mutant	25.20	7
Gatb	heterozygous	C57BL/6N	f	control	21.81	10
				mutant	22.16	9
			m	control	27.32	10
				mutant	26.30	9

	**Zygosity**	**Background**	**Sex**	**Genotype**	$\overline{bw}[g]$	**No.**
Gstm1	homozygous	C57BL/6N	f	control	21.76	8
				mutant	21.42	6
			m	control	26.83	8
				mutant	25.67	7
Kansl1l	homozygous	C57BL/6N	f	control	21.11	10
				mutant	20.97	10
			m	control	26.35	8
				mutant	24.96	8
Slc6a15	homozygous	C57BL/6N	f	control	23.59	16
				mutant	23.46	10
			m	control	27.90	16
				mutant	27.43	9
Sytl4	homozygous	C57BL/6N	f	control	21.84	10
				mutant	22.04	10
			m	control	27.03	10
				mutant	27.61	7
Uggt2	homozygous	C57BL/6N	f	control	21.73	23
				mutant	21.58	10
			m	control	26.40	21
				mutant	26.36	10
Zfp280d	homozygous	C57BL/6N	f	control	22.25	15
				mutant	21.75	8
			m	control	26.97	17
				mutant	23.98	10
