# Supplementary material for: Echo2Pheno: a deep-learning application to uncover echocardiographic phenotypes in conscious mice
Source: Mamm Genome. 2023 May 23;34(2):200–15. doi: 10.1007/s00335-023-09996-x (PMC10290584; doi:10.1007/s00335-023-09996-x)
Supplement: Supplementary file 2 — Supplementary captions (doc 13 KB) [file 335_2023_9996_MOESM2_ESM.docx]

**Supplementals:**

**Figure S1.** Confusion matrix showing true positive, false positive, false negative and true negative predictions on the test set of the quality assessment network.

**Figure S2.** Pearson correlations per study. Pearson correlations r with p for LVIDs (orange) and LVIDd (blue).

**Figure S3.** Boxplots per study. Left panel shows the distributions of LVIDs as boxplots and median per mouse in orange, the right one shows LVIDd as boxplots and median in blue.

**Figure S4.** Bland-Altman plots define the degree of method agreement for LVIDs (orange) and LVIDd (blue).

**Figure S5.** Bland-Altman plots per study. They define the degree of method agreement for LVIDs (orange) and LVIDd (blue).

**Table S1.** Wilcoxon-Rank-Sum-Test Results. Significant values are highlighted in orange.

**Table S2.** Metadata of the train and test datasets (f: female, m: male, bw: body weight).

**Table S3.** Metadata of the 16 validation studies (f: female, m: male, bw: body weight).
